# Developing the Workforce of the Digital Future: Leveraging Technology to Train Community-Based Mobile Mental Health Specialists

**DOI:** 10.1007/s41347-022-00270-6

**Published:** 2022-08-03

**Authors:** Benjamin Buck, Sarah L. Kopelovich, Justin S. Tauscher, Lydia Chwastiak, Dror Ben-Zeev

**Affiliations:** 1 Behavioral Research in Technology and Engineering (BRiTE) Center, Department of Psychiatry and Behavioral Sciences, University of Washington, Seattle, USA; 2 Supporting Psychosis Innovation through Research, Implementation and Training (SPIRIT) Lab, Department of Psychiatry and Behavioral Sciences, University of Washington, Seattle, USA

**Keywords:** mHealth, Digital health, Technology, Training, Implementation

## Abstract

Challenges in training, dissemination, and implementation have impeded the ability of providers to integrate promising digital health tools in real-world services. There is a need for generalizable strategies to rapidly train real-world providers at scale to support the adoption of digital health. This study describes the development of principles guiding rapid training of community-based clinicians in the support of digital health. This training approach was developed in the context of an ongoing trial examining implementation strategies for FOCUS, a mobile mental health intervention designed for people with serious mental illness. The SAIL (Simple, Accessible, Inverted, Live) model introduces how digital tools can be leveraged to facilitate rapid training of community agency-based personnel to serve as digital mental health champions, promoters, and providers. This model emphasizes simple and flexible principles of intervention delivery, accessible materials in a virtual learning environment, inverted or “flipped” live training structure, and live consultation calls for ongoing support. These initial insights lay the groundwork for future work to test and replicate generalizable training strategies focused on real-world delivery of digital mental health services. These strategies have the potential to remove key obstacles to the implementation and dissemination of digital health interventions for mental health.

## Introduction

Mental health care is in the midst of a digital transition (Ben-Zeev, 2020). Over the last decade, numerous digital technologies designed to assess, monitor, and treat mental illnesses have emerged (Van Ameringen et al., 2017), and the COVID-19 pandemic has only served to accelerate this growth (Torous et al., 2020b; Wind et al., 2020). During the pandemic, demand for digital health increased rapidly (Connolly et al., 2021), investors funneled billions of dollars to private sector digital mental health start-up companies (Ladka, 2021), and regulations were loosened to allow for technologies to support care continuity during the public health emergency (HHS Press Office, 2020). This growth has been fueled by tremendous unmet need for mental health services among individuals with serious mental illnesses (Walker et al., 2015). Publicly funded community behavioral health agencies in particular face crisis-level workforce shortages (Assistant Secretary for Preparedness and Response (ASPR), 2021; Health Resources and Services Association, 2021; Turale & Nantsupawat, 2021), and demand for services continues to increase in the wake of the COVID-19 pandemic (Vahratian et al., 2021). Digital health interventions present a promising means of addressing population mental health needs (Arevian et al., 2020; Friis-Healy et al., 2021; Rauschenberg et al., 2021); however, several barriers prevent this promise from being actualized in real-world settings (Mohr et al., 2018).

One crucial barrier to real-world implementation is the lack of a workforce trained to provide digital health interventions (Nemec & Chan, 2017; Torous & Keshavan, 2020). The effectiveness of unguided digital mental health interventions is limited by higher dropout rates relative to human supported digital interventions (Mohr et al., 2019, 2021; Torous et al., 2020a). Trained clinicians are a critical component to ensuring client engagement, understanding and continued functioning of digital mental health interventions, and supporting the development and maintenance of required digital infrastructure in clinical settings (Ben-Zeev et al., 2015; Graham et al., 2019; Schueller et al., 2017; Wisniewski & Torous, 2020). Trained personnel can provide ongoing motivational and technical support within the framework of “supportive accountability” (Mohr et al., 2011), wherein the interpersonal relationship with a caring facilitator increases intervention adherence. Although core competencies for mHealth supporting clinicians have been proposed (Schueller et al., 2021), to date, there has been little effort to establish efficient, scalable strategies for training the existing workforce in these core competencies.

To realize the promise of the growing body of empirical support for digital mental health interventions, strategies to rapidly and scalably train mental health staff to champion, promote, and deliver digital health interventions are required. We report here on four key principles that have grounded our efforts to train community-based behavioral health providers in the delivery of digital health. Our group is conducting a hybrid type III effectiveness-implementation trial across community mental health agencies across the state of Washington examining FOCUS — a mobile health (mHealth) intervention designed to support self-management in individuals with serious mental illness (SMI). Illness self-management describes empowering individuals with serious mental illnesses to monitor clinical status and use available resources or coping skills to respond accordingly to achieve or maintain recovery (Ben-Zeev et al., 2013; Mueser et al., 2006). The FOCUS program combines the use of a patient-facing mobile application with an mHealth Support Specialist (mHSS) who is a trained provider offering technical, motivational, and clinical support. FOCUS has demonstrated feasibility and acceptability among individuals with SMI (Ben-Zeev et al., 2014), similar clinical benefits to clinic-based evidence-based treatments (Ben-Zeev et al., 2018), cost-efficiency (Ben-Zeev et al., 2021), and high levels of engagement (Buck et al., 2020). Our ongoing study provided an opportunity to develop scalable digital mental health strategies. No previous deployments of FOCUS have involved training multiple community-based providers to serve as mHSS at multiple locations. Further, the constraints of the COVID-19 pandemic required that our group’s training activities take place entirely virtually. Here we describe key principles to guide digital mental health training, provide examples from the FOCUS mHealth effort, and discuss potential implications for meeting the challenge of rapidly training a digital-health enabled workforce.

### FOCUS mHealth Support Specialist Role and Tasks

The FOCUS mHealth support specialist (mHSS) role is informed by telebehavioral health competencies developed by the Coalition for Technology in Behavioral Science (CTIBS; (Maheu et al., 2017) and builds on previous work involving research deployments of FOCUS (Ben-Zeev et al., 2015; Jonathan et al., 2017). The mHSS has four key tasks: (1) preparing the clinic for FOCUS launch (e.g., raising awareness, training colleagues, establishing technological infrastructure), (2) installing the technology and orienting clients, (3) facilitating measurement-based care by synthesizing and sharing data with the clinical team, and (4) providing ongoing mHealth skills coaching to clients through weekly 10- to 15-min phone calls.

### Remote Training of the mHealth Support Specialist

The virtual training sequence is completed over a period of 2 weeks. It is anchored by two 4 h live trainings delivered via videoconferencing by an expert in mHealth clinical care (i.e., the “trainer”) to clinicians at community-based behavioral agencies who will fill the mHSS role (i.e., “trainees”). In advance of the first training week, trainees are provided introductory videos and written material that provide an initial primer and overview to the program (about 10 min of video, 5 pages of written text). Then, prior to each live training, trainees are requested to asynchronously and independently read and review relevant materials (each time above 30 min of video, and 15 to 20 pages of text) at least a week in advance (i.e., week #1 is used for preparation for training #1, week #2 preparation for training #2). One week before the first training, the trainer sends an email containing links to excerpts of the training manual and videos providing didactic content covering client-facing tasks (i.e., mHealth support calls and installing FOCUS). Following the first training, trainees receive links to videos and readings related to implementation-related tasks (e.g., sharing FOCUS data with the clinical team, and preparing the clinic for launch). Trainees are encouraged to practice client-facing tasks with peers in the week preceding second training (e.g., behavioral rehearsal in clinic or interacting with the FOCUS system), and the subsequent live training provides opportunities for review, practice, and discussion. Following the training, trainees are allowed access to all training materials through our training website, and ongoing support is provided through consultation calls.

## Four Guiding Principles: Simple, Accessible, Inverted, Live (SAIL)

**Fig. 1:**
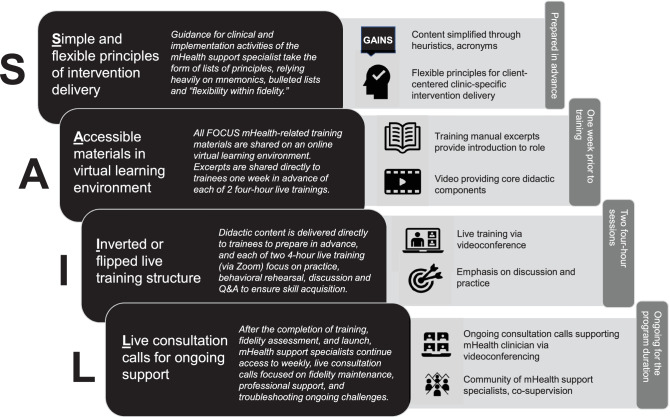
An overview of the specific components and principles of the SAIL model

Four principles ground this training approach, each of which we describe in the following sections and depict in Fig. 1: (1) simple and flexible principles of intervention delivery, (2) accessible online learning materials, (3) inverted or flipped live training structure, and (4) live consultation calls for ongoing clinical support. We believe these four principles—abbreviated as SAIL (Simple, Accessible, Inverted, Live)—have been key in allowing for a streamlined, remote, and technology-mediated training process and have potential to generalize to other healthcare systems introducing digital health. Importantly, these principles are flexible to the needs of specific training levels; individuals with Bachelor’s, Master’s and Doctoral levels of training have all received mHealth training that follows them, and overall materials are sufficiently flexible to match individual learner needs. In the sections to follow, we define each of the four key training principles, explain the rationale of each, and provide examples from FOCUS mHealth training to demonstrate.

### S: Simple and Flexible Principles of Intervention Delivery

First, the intervention model should be simple, flexible, and principle-driven. Treatment manuals introducing empirically supported treatments are often time-consuming to review and complex to implement (Stirman et al., 2010). Research on distance learning supports the use of abbreviated and focused content, reduced jargon, and mnemonics to allow for chunking and easier retention (Mahlangu, 2017). Further, the use of core principles—rather than protocolized directives—allows for flexible and patient-centered intervention delivery. This approach is consistent with the “flexibility within fidelity” (Kendall & Frank, 2018) framework, wherein core components (e.g., core intervention goals or coaching call components) are identified to allow for faithful implementation, but clinicians can present these within an “adaptable periphery,” responding to client needs, setting-specific characteristics and clinician strengths. This approach is particularly well-suited to digital health, where clinicians are redirecting their existing clinical skills toward novel applications rather than learning an entirely new set of professional activities.

FOCUS mHealth training takes this approach in content focused on structuring mHSS coaching calls with clients. Training in this task involves two mnemonics that outline both the structure and goals of a remote check-in session. The first—GAINS—describes how a session contains the following elements: *(1) Greeting and assessment*, where the mHSS gathers information about the client’s experience with the mHealth intervention, current symptoms, and goals for the call; *(2) Agenda*, where the mHSS structures the check-in call to follow; *(3) Interventions*, where the mHSS provides troubleshooting, motivational support, or personalization of self-management skills; *(4) Next steps*, where the mHSS and client agree to a plan for self-management, mHealth use or home practice; and *(5) Scheduling*, where the mHSS and client establish a plan for the next meeting.

The second mnemonic—TAPS—describe the key activities of an mHealth call: *(1) Technology troubleshooting* to ensure the device and app are functional for the client, *(2) Activation and motivation* to assist clients in building or maintaining motivation, *(3) Personalization of mHealth skills* to creatively adapt mHealth suggested content in a client-centered manner, and *(4) Summarization of interventions* to allow clients to synthesize the learning from the call. These elements are structured such that mHealth support specialists cover them in order of importance as time allows for a brief mHealth support call. For example, if a client’s device is not working, an entire call may focus on technology troubleshooting. In another instance, if the client has a functional device and is motivated to use the system, an entire call may focus on personalization of mHealth skills. GAINS and TAPS provide straightforward and memorable but flexible organizational principles to guide an mHealth coaching session. For example, while the mHSS is prepared to target all elements of the TAPS acronym, an individual mHealth call may focus on one targeted objective (e.g., application of a particular FOCUS skill or increasing motivation to use the intervention). Another call might be delivered out of order given immediate client needs (e.g., client activation or crisis) but still maintains all the core components of an mHealth call laid out by GAINS. For example, providers can revisit the call agenda if additional assessment reveals a need for a focus on particular set of issues in that call.

### A: Accessible Materials Through a Virtual Learning Environment

Second, materials must be accessible to trainees online. Increasingly, medical professionals across disciplines are reliant on and prefer digital resources (Egle et al., 2015). Digital materials are quickly searchable, shareable, and accessible across multiple settings and devices. Further, digital resources allow other clinicians providing back-up or coverage rapid access to the same materials that were used to train the lead mHealth specialist. A quick transfer of this information is particularly important in community mental health settings, where employee turnover is high and trained providers may be re-deployed to other roles (Brabson et al., 2020).

The accessibility principle is demonstrated through the FOCUS mHealth online resource library. This website provides access to several resources, including the training manual, training videos, suggested practices to improve mHealth coaching skills, “cheat sheet” checklists for clinical and implementation tasks, and the consultation call schedule. Because these materials are centrally located and digital, they can be instantly updated and disseminated at once (compared to paper versions that would need to be re-printed, mailed, and distributed). We have made those materials publicly available and we encourage readers to visit FOCUSmHealthTraining.org to access them.

### I: Inverted or Flipped Live Training Structure

Third, live trainings should involve “flipped” or “inverted” teaching. This approach describes a pedagogical strategy that allows trainees to review didactic content asynchronously, in advance of class time, such that they can use these skills to transform knowledge into skill acquisition (Gilboy et al., 2015). For example, in this approach, a course instructor might pre-record a lecture, require students to view this video in advance of class meeting, and reserve in-class time for synchronous demonstrations, discussion, or rehearsal. Conventional training techniques may be insufficient to train learners in the complex skills involved in evidence-based mental health practice (Beidas & Kendall, 2010), and flipped training approaches allow for a balance of didactic training and deeper experiential practice, feedback, and discussion. Synchronous trainings aim to address issues that could emerge in the future with clients and permit trainers to assess functional competencies before launch (Beidas et al., 2014).

The flipped approach is best exemplified in FOCUS trainings through the pre-workshop dissemination of FOCUS videos and use of behavioral rehearsal during synchronous training sessions. Brief videos (i.e., no longer than 10–20 min) introducing FOCUS skills are sent to trainees at least one week prior to its corresponding synchronous training session. With didactic content reviewed in advance, synchronous training sessions can provide a brief review of didactic content, but emphasize question and answer, discussion, and experiential practices to facilitate knowledge and skill acquisition. Trainees observe the trainer demonstrate specific skills, then practice together in real time to allow for proximal and behaviorally specific feedback from peers and the trainer. For example, when learning FOCUS installation, trainees watch and read didactic content related to installing FOCUS before having the chance to practice with the group and trainer in vivo via videoconferencing. Or, when learning mHealth coaching, trainees review core principles of sessions in advance allowing synchronous training to emphasize observing the trainer, as well as practicing in vivo during the training session, and exchanging feedback on performance.

### L: Live Consultation Calls for Ongoing Support

Last, implementation is supported with ongoing trainer consultation. Consultation describes ongoing learning opportunities provided by trainers typically external to the intervention setting (McLeod et al., 2018); it serves several functions including continued training and skill development, problem solving, clinical case application, and guided model adaptation (Nadeem et al., 2013). Participation in consultation is linked with adherence to fidelity standards, improved clinician skills, and maintenance of practices over time (Beidas et al., 2012). Group-based consultation also has the potential to provide the opportunity for community and peer support; these factors could be protective against burnout through increasing group cohesion and improving perceptions of the workplace atmosphere (Lasalvia et al., 2009).

Consultation is provided in FOCUS Training through weekly group consultation with a FOCUS trainer delivered through videoconferencing. Consultation meetings evenly emphasize both clinical (i.e., patient-facing) and implementation (i.e. clinic-facing) activities. The trainer sets an agenda weekly that includes (1) clinical case review, (2) implementation problem-solving, and (3) a pre-prepared “topic of the week” didactic presentation. Trainees share metrics related to intervention delivery (e.g., pacing of client recruitment, client engagement with FOCUS) and generate clinical and implementation topics to discuss. The trainer leads the group in discussion, troubleshooting, or behavioral rehearsal practice in the moment, and often, these discussions inform the subsequent week’s “topic of the week” didactic. When weekly discussion is completed, the trainer delivers the topical presentation, either through brief instructional materials (e.g., PowerPoint slides) or verbal instruction. Examples of training content reviewed in consultation include individual tailoring of FOCUS self-management skills, agenda setting in coaching calls, using data to inform and personalize mHealth coaching, and treatment termination. Calls take place weekly for 1 h and are available to mHealth support specialists regardless of their time in the role, number of previously attended consultation calls, or skill level. In addition to providing didactic content, discussion and practice opportunities, consultation calls have also provided opportunities for group support and camaraderie. For example, as new trainees have joined consultation calls, more senior specialists have informally provided mentorship and shared knowledge to support junior trainees.

## Limitations and Areas for Growth

Though our team has experienced how SAIL principles can facilitate successful and rapid training of an mHealth workforce, much remains to be learned in this area. First, little is known about relative efficacy of remote versus in-person training modalities for specific training purposes such as mHealth support roles, as well as the additive value of each particular component (e.g.,  online resource library vs. consultation calls and so on) in ensuring a well-trained workforce. Second, our training experiences have oriented around an intervention-specific mHealth support specialist role (i.e., the FOCUS mHealth support specialist), which differs from other models that have been suggested in the academic literature, such as digital navigators who provide guidance and support focused on multiple digital tools simultaneously (Wisniewski & Torous, 2020). Different models may suit different professionals, settings, and target populations. Third, while the hybrid training model provides some resource savings, it is not without costs; for example, the training website requires updates and maintenance. Future work is needed to address questions related to long-term cost and sustainability both of SAIL training and related implementation strategies. Our team’s ongoing hybrid type-III implementation/effectiveness trial will provide an important first step, and subsequent studies should continue to test the specific additive value of particular training components. Finally, while the combination of multiple elements of training and sustainment allows for personalization, much remains to be learned about how elements could be adapted to optimize training and consultation for the needs, preferences, and constraints of individual learners and settings.

## Conclusion

Our team has developed and successfully deployed a remote workforce development approach consisting of a hybrid (live and asynchronous) training delivered over 2-3 weeks, an online resource library, and ongoing consultation calls. Key principles of this strategy are captured within SAIL, as our approach has been (S) simple and principle-driven, with (A) accessible online learning materials, (I) inverted to allow for meaningful experiential learning, and with (L) live consultation calls to provide ongoing support. As mental health services continue to incorporate digital technologies to keep up with increasing demand, client preferences, and standards of care, pressures for healthcare systems to rapidly scale up a trained workforce will grow. This model provides an example of how a community mental health setting could go from having little to no experience with digital health interventions to having a provider well equipped and supported to provide digital mental health interventions in a matter of weeks.
